# Supplementary material for: In vivo crosslinking and effective 2D enrichment for proteome wide interactome studies
Source: Commun Chem. 2025 Aug 13;8:245. doi: 10.1038/s42004-025-01644-6 (PMC12350791; doi:10.1038/s42004-025-01644-6)
Supplement: Supplementary file 2 — Supplementary Information [file 42004_2025_1644_MOESM2_ESM.pdf]

In vivo crosslinking and effective 2D enrichment for proteome wide interactome studies.

Phillip Bräuer[1], Laszlo Tirian[3], Fränze Müller[1], Karl Mechtler[1,2,3, §], Manuel Matzinger[1,§]

[1] Research Institute of Molecular Pathology (IMP), Vienna BioCenter (VBC), Vienna, Austria.
[2] Gregor Mendel Institute of Molecular Plant Biology (GMI), Austrian Academy of Sciences, Vienna BioCenter (VBC), Vienna, Austria.
[3] Institute of Molecular Biotechnology (IMBA), Austrian Academy of Sciences, Vienna BioCenter (VBC), Vienna, Austria.

* These authors contributed equally
§ Correspondence to karl.mechtler@imp.ac.at, manuel.matzinger@imp.ac.at

Contents

Supplemental Figure 1: Benchmarking of Sepharose vs Magnetic bead material 2
Supplemental Figure 2: Distribution of crosslinked & monolinked peptides along the SEC gradient: 2
Supplemental Figure 3: Representative SEC chromatograms 3
Supplemental Figure 4: Score distribution of confident target-target XLs and target-decoy ratios 3
Supplemental Figure 5: Representative spectra from searches towards different database sizes 4
Supplemental Figure 6: Sequence coverage of DDX39A & B 5
Supplemental Figure 7: DDX39A/B abundance in K562 wt vs GFP-clone cells 6
Supplemental Figure 8: Abundance distribution of crosslinked proteins 7
Supplemental Figure 9: PPI network from whole K562 cells. 8

Supplemental Figure 1: Benchmarking of Sepharose vs Magnetic bead material

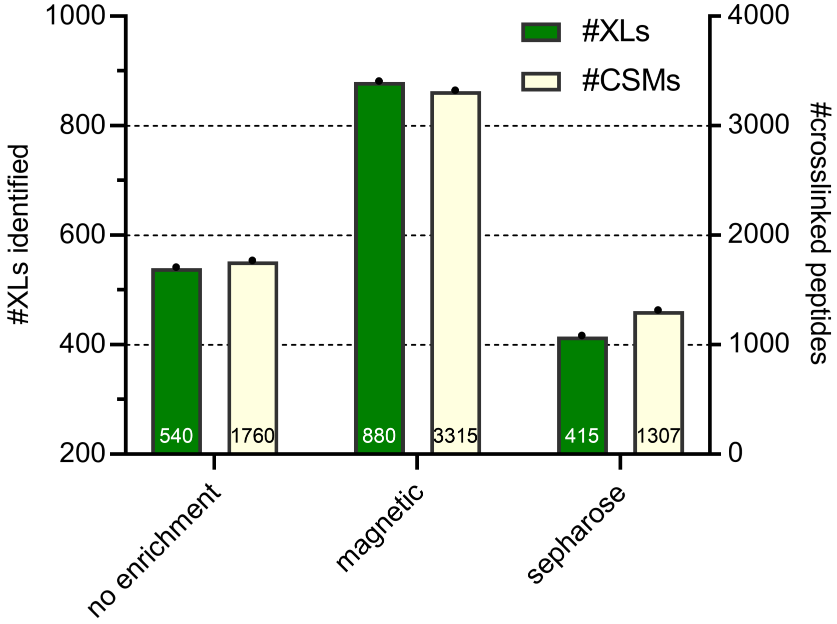

20 µg DSBSO crosslinked Cas9-Halo peptides were either enriched using Sepharose or magnetic beads (both Cytivia) using 30µL bead slurry and the exact same processing workflow. 800 ng of each enriched eluate or of a non-enriched control sample were subjected to LC-MS analysis using a 2 h active gradient on an Orbitrap Exploris. Data was searched against a database containing 117 proteins (Cas9 + crapome). Bars indicate identified unique crosslink sites or crosslink sequence matches at 1% FDR level, n=1.

Supplemental Figure 2: Distribution of crosslinked & monolinked peptides along the SEC gradient:

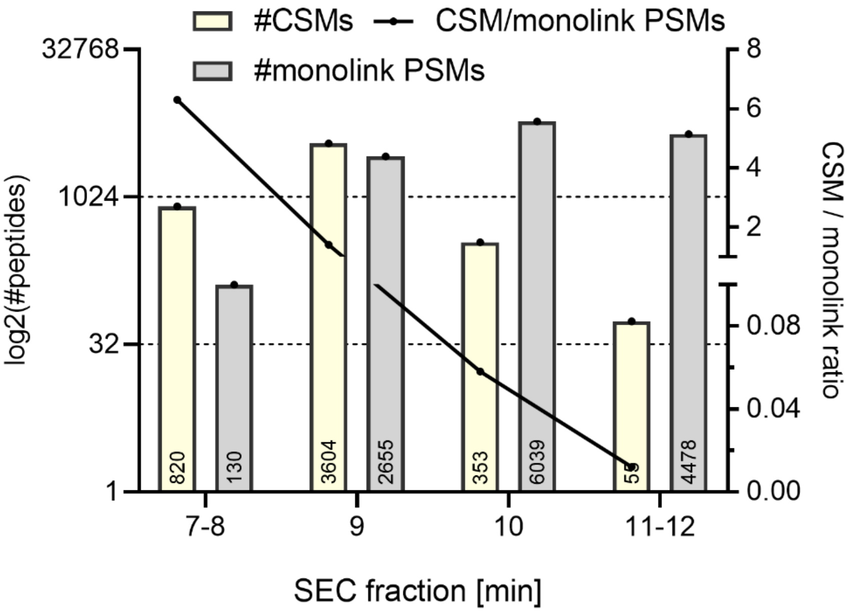

Data from Figure 3 D-F, representative replicate, whole K562 cells were DSBSO linked and enriched using magnetic DBCO beads followed by SEC. Fractions from minute 7-12 were analyzed by means of LC-MS. Data was searched against the human proteome. Bars indicate identified crosslinked peptide matches (CSM) or monolink PSM matches carrying a DSBSO modification at 1% FDR level, the line shows their ratio, n=1.

Supplemental Figure 3: Representative SEC chromatograms

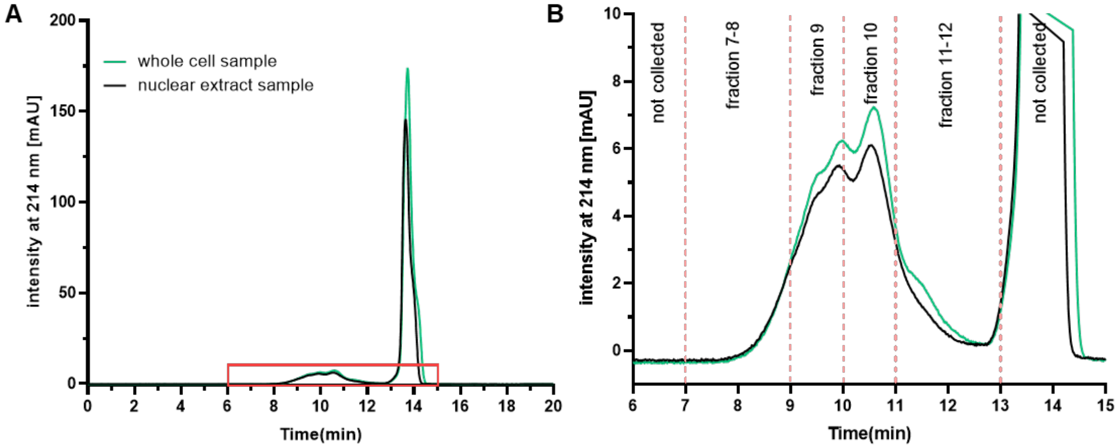

*Representative UV traces obtained from SEC from crosslinked samples after affinity enrichment obtained from whole cells or nuclear extracts as indicated. Shown is replicate 1 each, with resulting MS data as shown in Figure 4 A & B respectivley. SEC was performed using a TSKgel SuperSW2000 column at a flow rate of 0.3 mL/min with detection at 214 nm. The elution buffer consisted of isocratic elution with 30% (v/v) ACN in 0.1% (v/v) TFA in water. **A**: Full chromatogram, with red rectangle indication area zoomed into within B. **B**: Zoom into chromatogram with dashed red lines indication collected fractions.*

Supplemental Figure 4: Score distribution of confident target-target XLs and target-decoy ratios

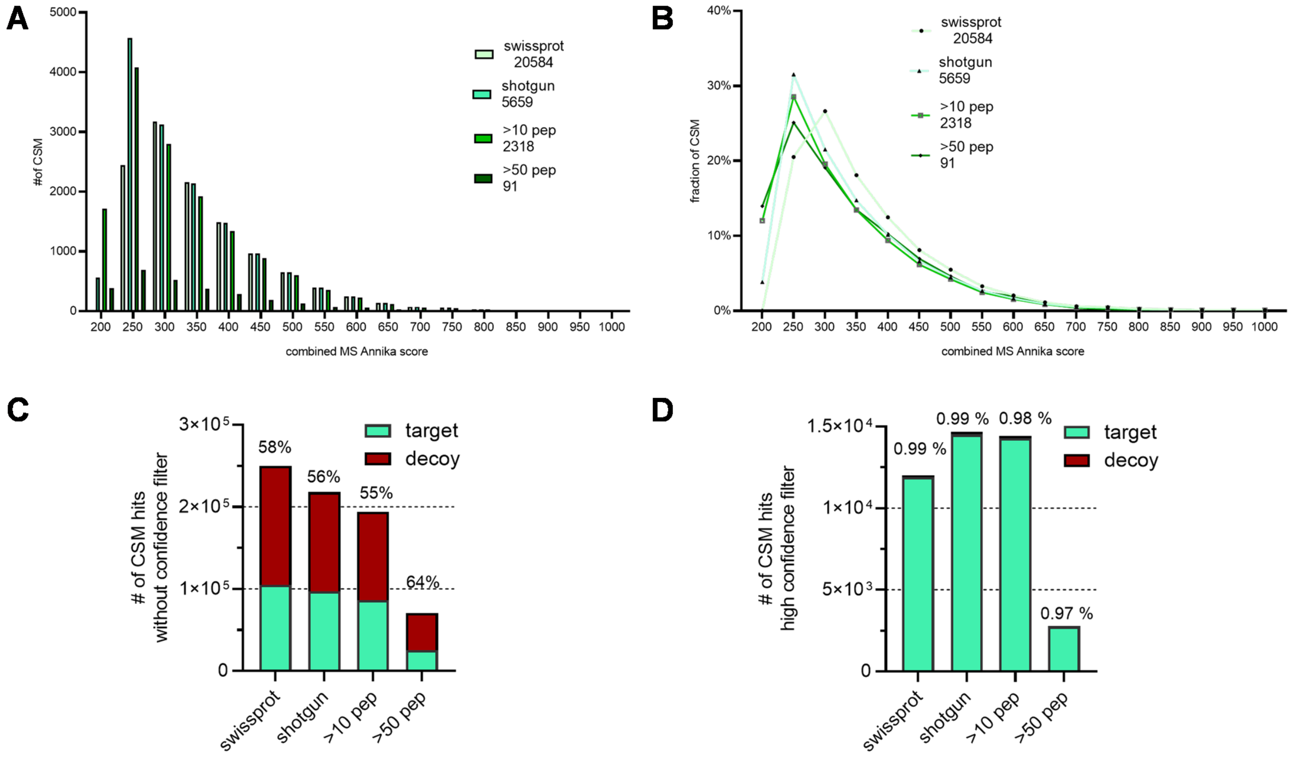

*Data from Figure 4 C, from whole cell K562 crosslinked samples searched towards databases of different size. **A** Histogram of all CSM matches vs their scores found and **B** relative numbers of CSM matches vs their scores found when searching towards databases of indicated sizes and after filtering for 1 % FDR on CSM level. **C**: All CSM matches without any confidence or FDR filter are shown reporting the distribution of decoy and target matches available prior to FDR calculation. **D**: Target and decoy CSM matches after FDR filtering to 1% done by MS Annika 3.0 are shown **C&D**: Relative fraction of decoy (sum of decoy-decoy, target-decoy or decoy-target links) is shown as number above each bar.*

Supplemental Figure 5: Representative spectra from searches towards different database sizes

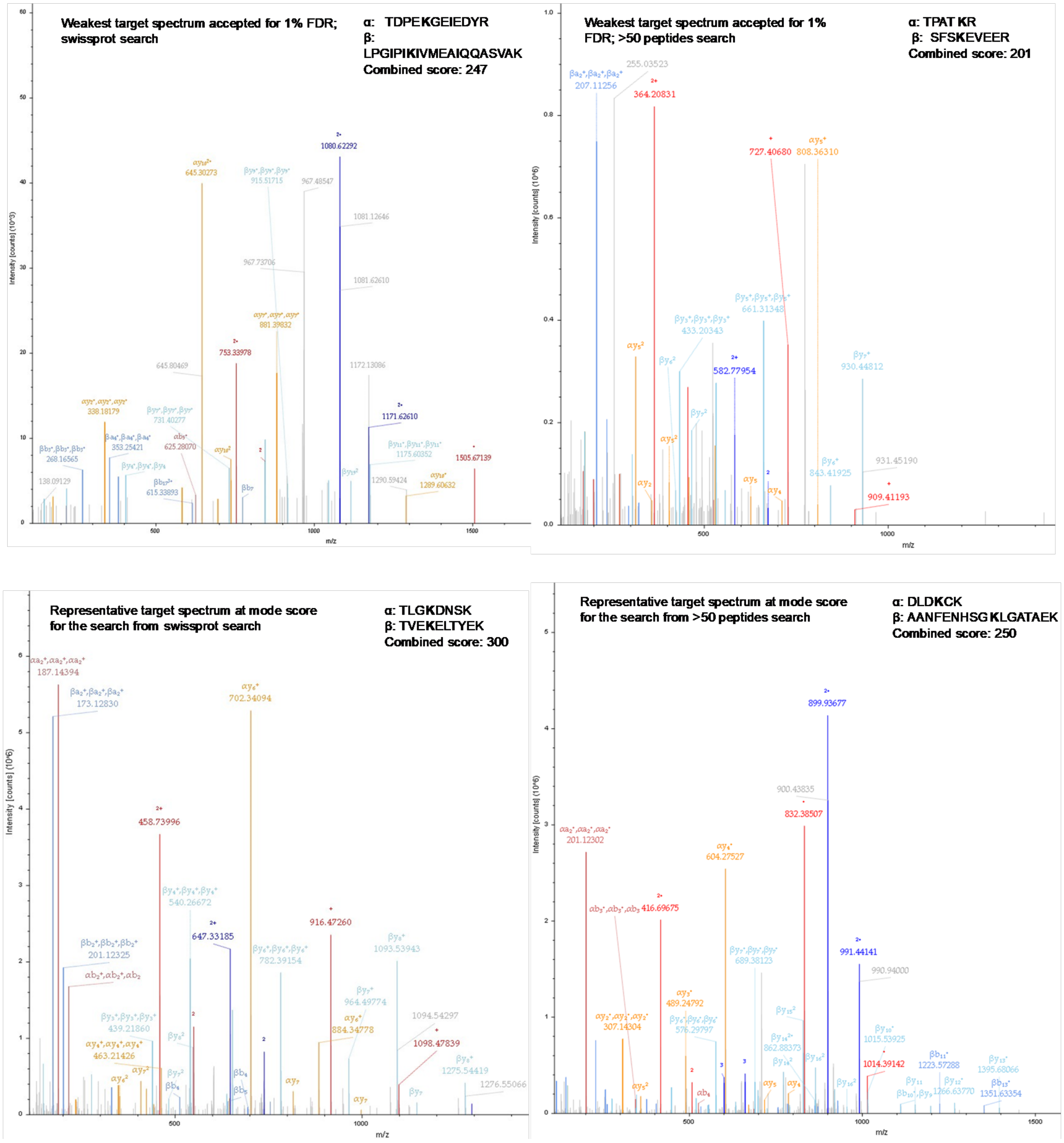

Data from Figure 4C. Left: Database search towards the full human swissprot containing 20584 sequences. Right: Database search towards a reduced shotgun database with proteins found with at least 50 peptides there. The database contains 91 sequences. Top: Annotated spectra as shown by Proteome Discoverer for the worst target CSM that was surpassing the 1% FDR threshold. Bottom: Representative annotated target spectra as shown by Proteome Discoverer for a CSM with the mode score within the score distribution for CSMs that surpassed the 1% FDR threshold.

Supplemental Figure 6: Sequence coverage of DDX39A & B

```
DDX39B  1     MAENDVDNELLDYEDDEVETAAGGDGAEAPAKKDVKGSYVSIHSSGFRDFLLKPELLRAI     60
              MAE  DV+N+LLDY  D+E  E  A  +          AP   KKD+KGSYVSIHSSGFRDFLLKPELLRAI
DDX39A  1     MAEQDVENDLLDY- DEEEEPQAPQESTP APP KKDI KGSYVSIHSSGFRDFLLKPELLRAI     59

DDX39B  61    VDCGFEHPSEVQHECIPQAILGMDVLCQAKSGMGKTAVFVLATLQQLEPVTGQVSVLVMC   120
              VDCGFEHPSEVQHECIPQAILGMDVLCQAKSGMGKTAVFVLATLQQ+EPV GQV+VLVMC
DDX39A  60    VDCGFEHPSEVQHECIPQAILGMDVLCQAKSGMGKTAVFVLATLQQIEPVNGQVTVLVMC   119

DDX39B  121   HTRELAFQISKEYERFSKYMPNVKVAVFFGGLSIKKDEEVLKKNCPHIVVGTPGRILALA   180
              HTRELAFQISKEYERFSKYMP+VKV+VFFGGLSIKKDEEVLKKNCPH+VVGTPGRILAL
DDX39A  120   HTRELAFQISKEYERFSKYMPSVKVSVFFGGLSIKKDEEVLKKNCPHVVVGTPGRILALV   179

DDX39B  181   RNKSLNLKHIKHFI LDECDKMLEQLDMRRDVQEIFRMTPHEKQVMMFSATLSKEIRPVCR   240
              RN+S + LK++KHF+LDECDKMLEQLDMRRDVQEIFR+ TPHEKQ   MMFSATLSK+IRPVCR
DDX39A  180   RNRSFSLKNVKHFVLDECDKMLEQLDMRRDVQEIFRLTPHEKQCMMFSATLSKDIRPVCR   239

DDX39B  241   KFMQDPMEI FVDDETKLTLHGLQQYYVKLKDNEKNRKLFDLLDVLEFNQVVIFVKSVQRC   300
              KFMQDPME+FVDDETKLTLHGLQQYYVKLKD+ EKNRKLFDLLDVLEFNQV+IFVKSVQRC
DDX39A  240   KFMQDPMEVFVDDETKLTLHGLQQYYVKLKDS EKNRKLFDLLDVLEFNQVIIFVKSVQRC   299

DDX39B  301   I  ALAQLLVEQNFPAIAIHRGMP QEERLSRYQQFKDFQRRILVATNLFGRGMDIERVNIAF   360
              + ALAQLLVEQNFPAIAIHRGM   QEERLSRYQQFKDFQRRILVATNLFGRGMDIERVNI   F
DDX39A  300   MALAQLLVEQNFPAIAIHRGMAQEERLSRYQQFKDFQRRILVATNLFGRGMDIERVNIVF   359

DDX39B  361   NYDMPEDSDTYLHRVARAGRFGTKGLAITFVSDENDAKILNDVQDRFEVNISELPDEIDI   420
              NYDMPEDSDTYLHRVARAGRFGTKGLAITFVSDENDAKILNDVQDRFEVN++ELP+EIDI
DDX39A  360   NYDMPEDSDTYLHRVARAGRFGTKGLAITFVSDENDAKILNDVQDRFEVNVAELPEEIDI   419

DDX39B  421   SSYIEQTR                                                       428
              S+YIEQ+R
DDX39A  420   STYIEQSR                                                       427
```

Gray: crosslinked peptide found unambiguous for DDX39B
Yellow: crosslinked peptide found ambiguous for DDX39A or B
Blue: crosslinked peptide found unambiguous for DDX39A
Bold: crosslink position, *italic*: cleavage site

Sequences of DDX39A (UniProt ID O00148) DDX39B (UniProt ID: Q13838) and their overlap. Sequences as used for our crosslink search with crosslinked peptides found unambiguous for either DDX39A or B or ambiguous for both highlighted as indicated.

Supplemental Figure 7: DDX39A/B abundance in K562 wt vs GFP-clone cells

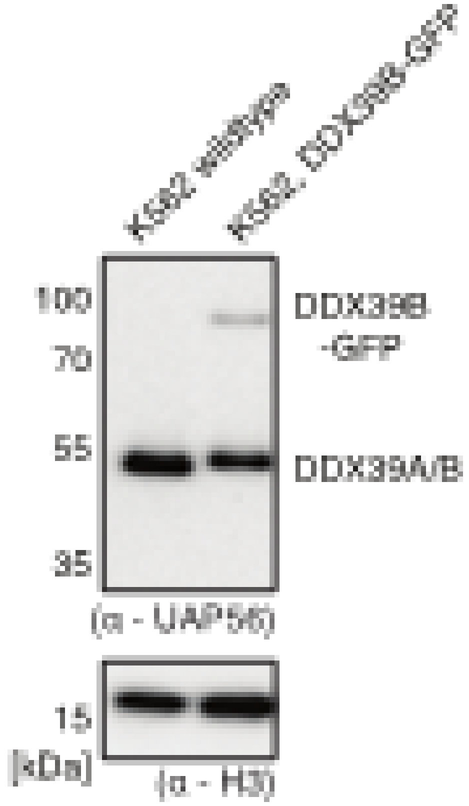

Immunoblot analysis of K562 wildtype and DDX39B-GFP clone cellular lysates, estimating relative abundances of DDX39 A/B using an antibody against UAP56 (~55 kDa, ~90 kDa GFP tagged version). H3 was used as loading control using an antibody against Histone H3.x

Supplemental Figure 8: Abundance distribution of crosslinked proteins

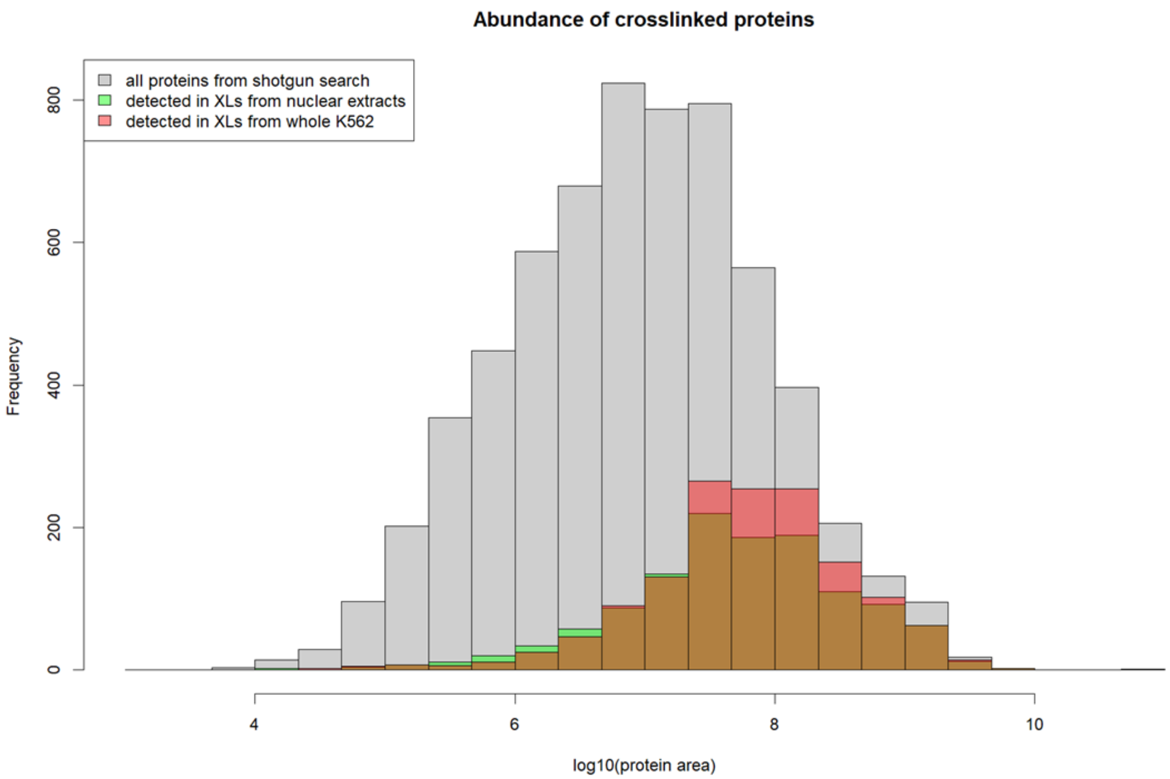

Histogram of log transformed relative abundance of all proteins found within n=5 replicates of a shotgun analysis from K562 matched to those proteins crosslinked within n=3 replicates of our nuclear extract or whole cell samples when searching them towards the database created from that shotgun analysis as shown in Figure 3 C & D. Of note, overlapping regions of green and red bars appear brown due to transparency.

Supplemental Figure 9: PPI network from whole K562 cells.

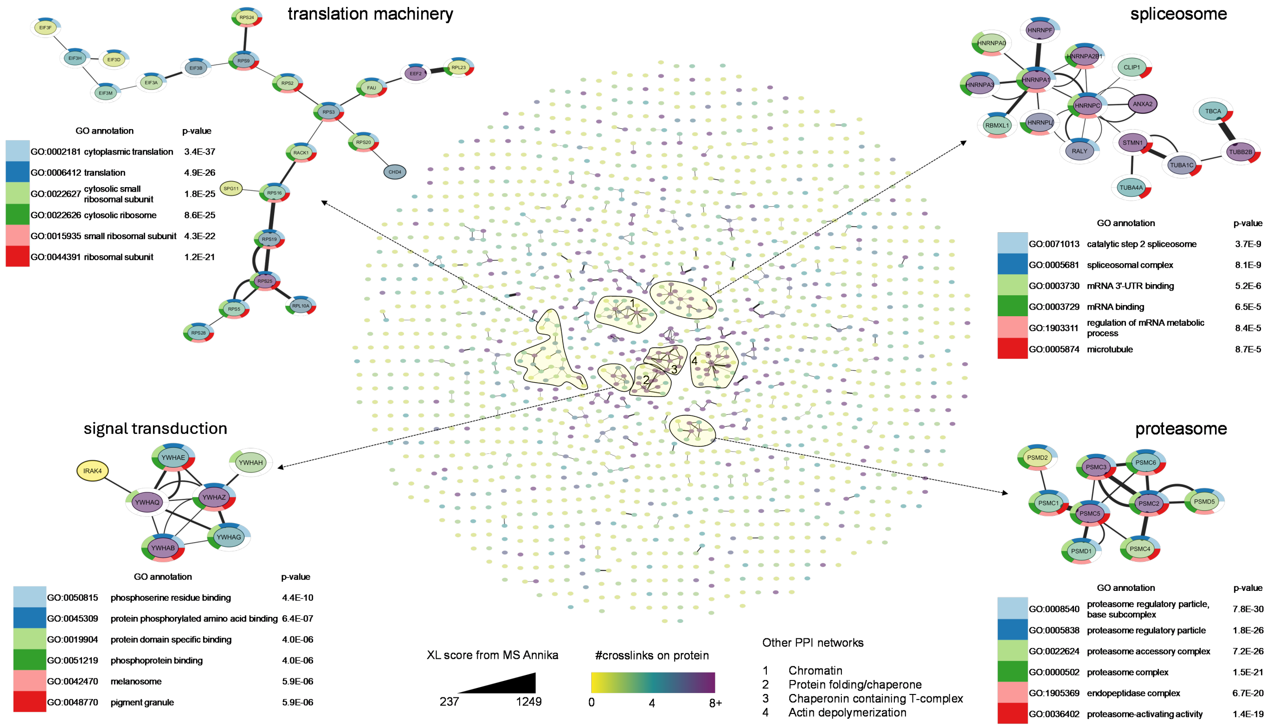

PPI network as found from intact cell crosslinking samples after analysis against a shotgun database (see Figure 3C) with correlated groups annotated. In total 311 non-ambiguous PPI from 610 heteromeric crosslinks were found. Selected sub-PPI-networks are highlighted with unambiguous inter-protein crosslinks shown. The edge thickness indicates the best MS Annika CSM score for each unique shown crosslink found within a 1% FDR threshold, with high scores indicating higher confidence. Node colors indicate the total number (inter- & intra-protein) of crosslinks found on each protein. The connected gene names are annotated for each protein and the top-6 gene-annotations after gene enrichment are shown.
