# Supplementary material for: In vivo crosslinking and effective 2D enrichment for proteome wide interactome studies
Source: Commun Chem. 2025 Aug 13;8:245. doi: 10.1038/s42004-025-01644-6 (PMC12350791; doi:10.1038/s42004-025-01644-6)
Supplement: Supplementary file 3 — Description of Additional Supplementary Files [file 42004_2025_1644_MOESM3_ESM.pdf]

Description of Additional Supplementary Files

File name: Supplementary Data 1

Description: Source data of all graphs including supplementary figures.

File name: Supplementary Data 2

Description: Predicted structure of native DDX39B

File name: Supplementary Data 3

Description: Predicted structure of DDX39B-FKB-GFP

File name: Supplementary Data 4

Description: Predicted structure of DDX39A/B dimer

File name: Supplementary Data 5

Description: Crosslinks from whole cell, shotgun DB

File name: Supplementary Data 6

Description: Crosslinks from nuclear extracts, shotgun DB

File name: Supplementary Data 7

Description: Novel crosslinks not in STRING, from nuclear extract, shotgun DB

File name: Supplementary Data 8

Description: AF predicted DDX39A + DDX39B dimeric structure
