# Supplementary material for: In vivo crosslinking and effective 2D enrichment for proteome wide interactome studies
Source: Commun Chem. 2025 Aug 13;8:245. doi: 10.1038/s42004-025-01644-6 (PMC12350791; doi:10.1038/s42004-025-01644-6)
Supplement: Supplementary file 12 — Reporting Summary [file 42004_2025_1644_MOESM12_ESM.pdf]

nature portfolio

Corresponding author(s): Manuel Matzinger, Karl Mechtler

Last updated by author(s): Jul 23, 2025

Reporting Summary

Nature Portfolio wishes to improve the reproducibility of the work that we publish. This form provides structure for consistency and transparency in reporting. For further information on Nature Portfolio policies, see our Editorial Policies and the Editorial Policy Checklist.

Statistics

For all statistical analyses, confirm that the following items are present in the figure legend, table legend, main text, or Methods section.

n/a	Confirmed	
☐	☒	The exact sample size (*n*) for each experimental group/condition, given as a discrete number and unit of measurement
☐	☒	A statement on whether measurements were taken from distinct samples or whether the same sample was measured repeatedly
☒	☐	The statistical test(s) used AND whether they are one- or two-sided<br>*Only common tests should be described solely by name; describe more complex techniques in the Methods section.*
☒	☐	A description of all covariates tested
☒	☐	A description of any assumptions or corrections, such as tests of normality and adjustment for multiple comparisons
☒	☐	A full description of the statistical parameters including central tendency (e.g. means) or other basic estimates (e.g. regression coefficient) AND variation (e.g. standard deviation) or associated estimates of uncertainty (e.g. confidence intervals)
☒	☐	For null hypothesis testing, the test statistic (e.g. *F*, *t*, *r*) with confidence intervals, effect sizes, degrees of freedom and *P* value noted<br>*Give P values as exact values whenever suitable.*
☒	☐	For Bayesian analysis, information on the choice of priors and Markov chain Monte Carlo settings
☒	☐	For hierarchical and complex designs, identification of the appropriate level for tests and full reporting of outcomes
☒	☐	Estimates of effect sizes (e.g. Cohen's *d*, Pearson's *r*), indicating how they were calculated

Our web collection on statistics for biologists contains articles on many of the points above.

Software and code

Policy information about availability of computer code

Data collection	Mass spectrometry data was acquired using the Orbitrap Exploris 480 or Eclipse MS, using Thermo Tune software (version: 0.4 or higher).
Data analysis	Proteomics data was analysed using MS Annika 3.0 for crosslinked peptides and Amanda 3.0 for linear peptides within Proteome Discoverer 3.1.<br>Relative, label free, quantification was done using apQuant3. Results were filtered to 1% FDR at peptide, protein, CSM and unique XL site level. A DSBSO linker based workflow as recommended in the most recent MS Annika publication (download: https://github.com/hgb-bin-proteomics/MSAnnika/raw/master/workflows/PD3.0/DSBSO_MS2.pdAnalysis) was used for data analysis.<br>For data visualization and statistics Graph Pad Prism 8.0 (GraphPad Software Inc.) was used. Venn diagrams were plotted using DeepVenn. Post processing to visualize 3D models was performed using xiVIEW, for proteome wide interaction network visualization and gene enrichment analysis Cytoscape v3.10.3 35 was used. Results obtained from Annika in Proteome discoverer were exported to xiview using a custom Python script available on GitHub (https://github.com/hgb-bin-proteomics/MSAnnika_exporters/blob/develop/xiViewExporter_msannika.py). 3D structure prediction was performed using AlphaFold3 on a local cluster. AlphaFold2 on a Google Colab sheet was used to predict the dimeric DDX39A-B structure, or using DDX39 A/B – CHTOP structures using the suggested default settings (https://colab.research.google.com/github/sokrypton/ColabFold/blob/main/AlphaFold2.ipynb?pli=1#scrollTo=R_AH6JSXaeb2). DDX39 A/B – CHTOP structures were further predicted including crosslink data from unambigous links found within this study, using AlphaLink2 on a Google Colab sheet (https://colab.research.google.com/github/Rappsilber-Laboratory/AlphaLink2/blob/main/notebooks/alphalink2.ipynb#scrollTo=j-xTD0QubEN-)

For manuscripts utilizing custom algorithms or software that are central to the research but not yet described in published literature, software must be made available to editors and reviewers. We strongly encourage code deposition in a community repository (e.g. GitHub). See the Nature Portfolio guidelines for submitting code & software for further information.

Data

Policy information about availability of data

All manuscripts must include a data availability statement. This statement should provide the following information, where applicable:

- Accession codes, unique identifiers, or web links for publicly available datasets
- A description of any restrictions on data availability
- For clinical datasets or third party data, please ensure that the statement adheres to our policy

The mass spectrometry raw proteomics data, Proteome Discoverer search results and used fasta files have been deposited to the ProteomeXchange Consortium via the PRIDE partner repository with the dataset identifier PXD061173. MS Amanda 3.0 (https://github.com/hgb-bin-proteomics/MSAmanda) and MS Annika 3.0, related workflow templates used for data analysis (https://github.com/hgb-bin-proteomics/MSAnnika) as well as code to generate XiView compatible exports from Annika data (https://github.com/hgb-bin-proteomics/MSAnnika_exporters/blob/develop/xiViewExporter_msannika.py are available on GitHub. Source data of all figures is provided as Supplementary Dataset.

Research involving human participants, their data, or biological material

Policy information about studies with human participants or human data. See also policy information about sex, gender (identity/presentation), and sexual orientation and race, ethnicity and racism.

Reporting on sex and gender	n.a.
Reporting on race, ethnicity, or other socially relevant groupings	n.a.
Population characteristics	n.a.
Recruitment	n.a.
Ethics oversight	n.a.

Note that full information on the approval of the study protocol must also be provided in the manuscript.

Field-specific reporting

Please select the one below that is the best fit for your research. If you are not sure, read the appropriate sections before making your selection.

☒ Life sciences ☐ Behavioural & social sciences ☐ Ecological, evolutionary & environmental sciences

For a reference copy of the document with all sections, see nature.com/documents/nr-reporting-summary-flat.pdf

Life sciences study design

All studies must disclose on these points even when the disclosure is negative.

Sample size	To assess technical variability, each experiment was performed using at least three independent replicates. No sample size calculation was performed. As we saw a low variance between these replicates aiming to check on the reproducibility of the workflows themselves, we decided that no further replicates were needed.
Data exclusions	Results were excluded from further downstream analysis in case of obvious failure (i.e. column overpressure during measurement, or other known errors leading to 0 or close to 0 IDs)
Replication	All experiments were performed using 3 replicates of which all were successful
Randomization	Not applicable, as we do not investigate biological effects and therefore had no groups. Replicate MS measurements of benchmark studies where measured in alternating order to avoid effects originating from altered MS performance with time.
Blinding	As this is not a clinical study, blinding is not applicable.

Reporting for specific materials, systems and methods

We require information from authors about some types of materials, experimental systems and methods used in many studies. Here, indicate whether each material, system or method listed is relevant to your study. If you are not sure if a list item applies to your research, read the appropriate section before selecting a response.

Materials & experimental systems

n/a	Involved in the study
☐	☒ Antibodies
☐	☒ Eukaryotic cell lines
☒	☐ Palaeontology and archaeology
☒	☐ Animals and other organisms
☒	☐ Clinical data
☒	☐ Dual use research of concern
☒	☐ Plants

Methods

n/a	Involved in the study
☒	☐ ChIP-seq
☒	☐ Flow cytometry
☒	☐ MRI-based neuroimaging

Antibodies

Antibodies used: Abcam ab181059 against UAP56 and Abcam ab1791e against Histone H3

Validation: Validated by the manufacturer

Eukaryotic cell lines

Policy information about cell lines and Sex and Gender in Research

Cell line source(s): K562 cells were obtained from DMSZ (https://www.dsmz.de/collection/catalogue/details/culture/ACC-10)

Authentication: None of the cell lines were authenticated.

Mycoplasma contamination: Used cells were tested negative of mycoplasma contamination.

Commonly misidentified lines (See ICLAC register): no commonly misidentified lines were used

Plants

Seed stocks: none

Novel plant genotypes: none

Authentication: none
